# Experimentally derived beam quality correction factors for PPC05 and razor nano ionization chambers in proton scanned beams based on graphite calorimetry

**DOI:** 10.1002/acm2.70860

**Published:** 2026-10-14

**Authors:** Nicolás Gómez‐Fernández, Séverine Rossomme, Eloy González‐García, Lies Verpoest, Faustino Gómez, Alejandro Mazal, Juan Antonio Vera‐Sánchez, Diego M González Castaño

**Affiliations:** ^1^ Radiation Physics Laboratory, AII University of Santiago de Compostela Santiago de Compostela Spain; ^2^ Clinical Department, Dosimetry Ion Beam Applications Louvain‐la‐Neuve Belgium; ^3^ Institut de Recherche Expérimentale et Clinique‐Molecular Imaging Radiotherapy and Oncology (MIRO) UCLouvain Louvain‐la‐Neuve Belgium; ^4^ Departamento de Física de Partículas Universidade de Santiago de Compostela Santiago de Compostela Spain; ^5^ Centro Protonterapia QuironSalud Pozuelo de Alarcón Spain; ^6^ Fundación de Investigación Sanitaria de Santiago Santiago de Compostela Spain

**Keywords:** beam quality correction factors, calorimetry, Protontherapy reference dosimetry

## Abstract

**Background:**

Ionization chamber based reference dosimetry of proton beam requires the use of beam quality correction factors which are obtained experimentally or by Monte Carlo simulation and are available to users in codes of practice and other publications.

**Purpose:**

The aim of this work is to determine experimental proton beam quality correction factors, $k_{Q}$, for two ionization chambers using a portable graphite calorimeter: the IBA reference‐class PPC05 chamber and the IBA non reference‐class Razor Nano chamber, using a portable graphite calorimeter.

**Methods:**

Measurements were performed in scanned clinical proton beams at two clinical centers. Absorbed dose to graphite was determined using a recently developed portable graphite calorimeter. The conversion factors for the absorbed dose to water determination were obtained by Monte Carlo simulations. Finally, the experimental $k_{Q}$ factors were obtained by comparing ionization chamber readings with the calorimeter, measured using the same conditions, in terms of absorbed dose to water.

**Results:**

For the IBA PPC05 chamber, the experimental values agreed with recommended values tabulated in TRS‐398 (rev. 1) within the combined experimental uncertainty for the three residual ranges of 3.4, 13.3 and 27.7 g·cm^−2^. For Razor Nano chamber, $k_{Q}$ values of 1.009 and 1.011 for residual ranges of 13.3 and 27.7 g·cm^−2^ have been determined. No recommended values are provided in TRS_398 (rev. 1). The overall budget yielded a combined standard uncertainty in the experimental $k_{Q}$ determination of less than 1% (k=1).

**Conclusions:**

This study demonstrates the feasibility and high precision of using a portable graphite calorimeter for beam quality correction factor determination in the user beam quality. The short room occupation time required (around 3 hours) makes the system useful as well for beam output dosimetry audits without disturbing clinical practice.

## 1 INTRODUCTION

As in every other radiotherapy modality, accurate determination of absorbed dose to water at reference irradiation conditions is essential for the overall accuracy of proton therapy treatments. Absorbed dose to water‐based formalisms, such as that described in the IAEA TRS‐398 Code of Practice,^1^ are based on calibration coefficients in terms of absorbed dose to water $N_{D,w,Q_{0}}$ determined in a Standard Dosimetry Laboratory at a reference beam quality (typically *^60^Co*). The application of these coefficients to the user beam qualities (*Q*) requires the introduction of beam quality correction factors, $k_{Q,Q_{0}}$ (or simply $k_{Q}$ if the reference beam quality is cobalt‐60). Therefore, the absorbed dose to water using an ionization chamber is determined by the following equation:

(1) $$D_{w,Q}=M_{Q}\;N_{D,w,Q_{0}}k_{Q,Q_{0}}$$

where $M_{Q}$ is the reading of the ionization chamber corrected for influence quantities (i.e., temperature and pressure, polarity and recombination). Although the availability of these beam quality correction factors for commercial ionization chambers has traditionally relied on semi‐empirical estimations and more recently on Monte Carlo numerical simulations,^2^, ^3^, ^4^, ^5^ there is a critical need for accurate experimental determinations to independently validate these theoretical models and minimize the associated clinical uncertainties.

Calorimetry stands as the most rigorous direct method for the absolute determination of absorbed dose. Although water calorimetry constitutes the gold primary standard for absorbed dose to water, its use in clinical centers is cumbersome given their level of complexity (bulkiness, need for cryogeny and gas supply). This is of special relevance in protontherapy centers, where access is more limited than in photon therapy. Nevertheless, there are some examples of portable water calorimeters employed in proton beam dosimetry,^6^, ^7^, ^8^, ^9^, ^10^, ^11^ although not until recently have been used in multi‐energy and multi‐chamber studies.^12^, ^13^ The development of portable graphite calorimeters has emerged as an efficient alternative for in situ reference dosimetry.^14^, ^15^ Being a solid and homogeneous material, graphite allows for the design of robust systems with high thermal stability, which optimizes room time without substantially interfering with the clinical routine of hospital centers. Nevertheless, the use of graphite requires the introduction of conversion factors from absorbed dose to graphite to absorbed dose to water, $C_{g,w}$, calculated analytically or by Monte Carlo simulations^16^ and therefore overall uncertainty may be higher than that of water calorimeters. General concepts of absorbed dose calorimetry, including application to proton beams can be found elsewhere.^17^

Recently, [REDACTED] has developed and validated a portable graphite calorimetry system^18^ designed specifically to minimize treatment room downtime (approximately 3 hours, including setup up, thermal stabilization, and measurements), achieving excellent thermal stability over hours (±100μK) and demonstrating outstanding agreement in inter‐comparisons of photon beam dosimetry against the primary standard of the National Physical Laboratory (NPL).^18^

The present work aims to test the performance of this portable calorimeter in proton beams and to expand data on experimental proton beam quality correction factors, $k_{Q}$, for a plane parallel ionization chamber widely used in clinical practice and to derive $k_{Q}$ factors for a small active volume cylindrical chamber for which there is no published data.

## 2 MATERIALS AND METHODS

### 2.1 Equations

Beam quality correction factors for an ionization chamber at a certain reference depth and for proton beam reference irradiation conditions, “*Q*”, can be derived from the quotient:

(2) $$k_{Q}=\frac{N_{D,w,Q}}{N_{D,w,Q_{0}}}\;$$

where $N_{D,w,Q_{0}}$ is the chamber calibration factor in terms of absorbed dose to water, determined at a Standards Dosimetry Laboratory for a reference beam quality (in a cobalt 60 beam) and $N_{D,w,Q}$ would be the calibration coefficient in terms of absorbed dose to water for the same chamber and for the user beam quality which is generally not available but for photon beam qualities in certain calibration laboratories. If we make $Q_{0}=Q$ in equation (1) we see that calibration coefficient for beam quality, $\;N_{D,w,Q}$, can be obtained by measuring the ratio:

(3) $$N_{D,w,Q}=\frac{D_{w,Q}}{M_{Q}}\;$$

where $D_{w,Q}$ is the absorbed dose to water at reference depth, measured by a reference detector of accuracy better than the user instrument, and $M_{Q}$ is the charge collected by the ionization chamber with its reference point placed at reference depth and corrected by influence quantities, whenever both are measured for the same delivered radiation beam.

### 2.2 Irradiation conditions

In this work we used four mono‐energy layer proton scanned beams delivered by an IBA ProteusOne system (see Table 1). For all measurements, the surface of the PMMA tank, in which the calorimeter (see section c) or the chambers (see section d) were positioned, was placed at isocenter. Measurements were performed in two clinical centers (Center 1: Rutherford Cancer Center, Reading, United Kingdom; Center 2: Centro de Protonterapia Quirónsalud, Madrid, Spain).

**TABLE 1:** Summary of irradiation conditions. Spot size refers to Qs which represents full width at 1/e^2^ of maximum of the Gaussian spatial distribution of the beam.

Energy (MeV)	Practical Range (g·cm^2^)	Center	spot spacing (mm)	*map* (X x Y spots)	Spot size $Q_{s}$ (mm)	Ion chamber
100	7.74	1	4	13 × 13	5.8	PPC05#475 PPC05#1496
	7.71	2	4	14 × 14	5.8	PPC05#1277
160	17.61	1	3	18 × 18	4.21	PPC05#475 PPC05#1496 RAZOR NANO#17475
	17.58	2	3	18 × 18	4.21	PPC05#1277
	17.58	2	2.5	41 × 41	4.21	PPC05#1277
226	32.04	1	2.5	21 × 21	3.65	PPC05#475 PPC05#1496 RAZOR NANO#17475
	31.97	2	2.5	21 × 21	3.65	PPC05#1277

The calorimeter was positioned, the Proportional Integral Derivative (PID) controller activated and once working temperature stabilized, several single energy layer scanned beams of varying sizes and energies (see Table 1) were delivered between 3 and 7 times each. Thus, absorbed dose to graphite was measured at core position for each single energy layer scanned beam. Absorbed dose to water was derived from these measurements by conversion factors (see section c.ii). For the ionometry part, the graphite calorimeter was extracted from the PMMA walled tank in which it is placed and replaced by the tested ionization chamber (see Table 1) filling this tank with water.

At center 1, calorimeter measurements were performed delivering 4 times more monitor units per spot than chamber measurements, while at center 2 both measurements were performed at the same monitor units per spot. All results were normalized to the reading of monitor chamber of the proton therapy system. Each single layer mono‐energy map is delivered in three bursts because of the limitations of the synchro‐cyclotron.^19^

### 2.3 Determination of absorbed dose to water

#### 2.3.1 Portable graphite calorimeter

Using our portable graphite calorimeter, we have derived absorbed dose to water from the measurement of absorbed dose to graphite by using:

(4) $$D_{w}^{\mathrm{Cal}}=c_{g}\;\Delta TC_{g,w}k_{\mathrm{hl}}k_{\mathrm{imp}}$$

where $c_{g}$ is the specific heat capacity of graphite, ΔT is the temperature rise due to the ionizing radiation beam delivery, $k_{hl}$ is a correction for heat losses, $k_{imp}$ is a correction for impurities present in the core and $C_{g,w}$ is the dose to graphite to dose to water conversion factor, obtained by Monte Carlo simulation for the specific radiation beam used during the measurement. This factor converts absorbed dose to graphite measured at the calorimeter core to absorbed dose to water in a small volume of water placed at the calorimeter core water equivalent depth (Other authors have recently denoted this factor as $f_{Q}$ ^20^). More details related to Monte Carlo simulations are provided in the next sub‐section. In equation (4), the product $c_{g}\Delta Tk_{hl}k_{imp}$ equals absorbed dose to graphite averaged in the calorimeter core or $D_{g}$. The net temperature increments (ΔT) in the calorimeter core were analytically determined for each irradiation series by calculating the difference between linear extrapolation of the core temperature slopes recorded during the pre‐drift and post‐drift periods at the middle time point of the irradiation. A detailed description of the temperature calibration of the embedded thermistors in the core can be found elsewhere.^18^ The sequence for measuring each irradiation map takes approximately two minutes. The first step consists of measuring the pre‐irradiation drift for 20 seconds. The beam is then turned on, and the radiation‐induced temperature rise in the calorimeter core is measured. Subsequently, the post‐ irradiation drift is determined for 20 seconds. Finally, the PID controller is allowed to operate for approximately 60 seconds to correct potential temperature deviations before repeating the measurement cycle. These times are adequate for the proper system operation as has been previously shown.^18^ Heat loss correction was assumed to be equal to the value obtained in a previous work for a radiation field size of equal area.^18^ Correction due to presence of impurities within the core was calculated for proton beams in the range 100 to 226 MeV by Monte Carlo simulation, using the same approach as in a previous work^18^ but with FLUKA Monte Carlo code (FLUKA2025 Version 1.1).^21^

The calorimeter core (a 2.33 mm thin graphite disk with embedded thermistors) is placed with its center at a physical depth of 2.8 cm in graphite (including air gaps) and the calorimeter is placed inside a PMMA walled container with an entrance window of 0.5 cm, resulting in a total physical depth from the external wall of 3.3 cm. For proton beam energies in the range 100 to 226 MeV, total water equivalent thickness calculated as described in a previous work^22^ is 4.3 cm.

A more detailed explanation on the calorimeter operation and how to obtain its operating parameters can be found elsewhere.^18^

#### 2.3.2 Monte Carlo model

The conversion factor needed for obtaining absorbed dose to water from calorimeter measurements can be evaluated by calculating the ratio:

(5) $$c_{g,w}\;\left(z_{w},\mathrm{map},E\right)=\frac{D_{w,\mathrm{MC}}\left(z_{w},\mathrm{map},E\right)}{D_{g,\mathrm{cal},\mathrm{MC}}\left(\mathrm{map},E\right)}\;$$

where $D_{w,MC}$ is the absorbed dose to a small volume of water at depth in water $z_{w}$ and $D_{g,cal,MC}$ is the absorbed dose to graphite scored at the calorimeter core, both simulated with FLUKA for a single layer proton beam of energy E and with a spot distribution denoted as map, which is defined by parameters spot size, spot spacing and field size. Simulation of absorbed dose to water comprises a water tank and simulation of absorbed dose to graphite the full calorimeter geometry. For both cases, each map is simulated by adding the individual results of each beamlet in the map. Since the detailed geometry of the calorimeter and the transport of radiation is included in the simulation, our conversion factor $C_{g,w}$ includes stopping power differences between water and graphite, correction for the presence of gaps, fluence perturbation, etc.

Validation of our Monte Carlo model comprises comparing pristine beam experimental and Monte Carlo simulated IDDs in water. For each of the measured energies, its momentum distribution, beam divergence and shape (described as Gaussian) were adjusted to reproduce experimental curves (provided by each center and measured with an IBA Stingray chamber). Ionization potentials for air, water, and graphite used in the simulations were 85.7 eV, 78 eV, and 81 eV, respectively, as recommended by ICRU Report 90.^23^

As an additional check of the Monte Carlo calculated relative depth doses for full maps, we compared absorbed dose to water measured with a PPC05 chamber at depths of 2, 5 and 10 cm (for 100, 160 and 226 MeV respectively) with absorbed dose to water measured with the calorimeter at its reference depth and converted to the aforementioned depths by appropriate Monte Carlo factors, calculated by using equation (5) with $z_{w}$ = 2, 5 or 10 cm. This was done at “center 2” for 5 cm x 5 cm maps (for all energies) and for a 10 cm x 10 cm map (for 160 MeV) during a separate experimental session.

### 2.4 Ion chamber measurements

Three IBA dosimetry PPC05 (SN 475, 1277 and 1496) and an IBA dosimetry Razor Nano (SN 17475) ionization chambers were used. All chambers were calibrated in terms of absorbed dose to water in cobalt 60 reference beam quality at [REDACTED], against an ionometric secondary standard calibrated at Physikalisch Technische Bundesanstalt.

The effective point of measurement of chambers was positioned at the same water equivalent depth (i.e., 4.3 cm) as the effective point of measurement of the graphite calorimeter. The chambers were positioned according to TRS‐398 recommendations^1^: For the PPC05 chamber the effective point of measurement is the center of the inner surface of the entrance electrode. For the Razor Nano the effective point of measurement is the middle of the spherical cavity. For each chamber, collected charge was measured using the same beam configuration as that used with the graphite calorimeter.

Voltages of operation were 500 V for PPC05 chambers and 300 V for Razor Nano chamber. An IBA DOSE‐1 electrometer was used for polarization and charge readout. Saturation correction factors were measured for all chambers at least in one of the three energies. For evaluating the saturation correction factor, we followed TRS‐398 recommendations and derived the factors from the linear fit of the inverse of charge versus inverse of polarization voltage (Jaffé plot), itself a method based in applying Boag theory to pulsed beams. Linearity was evaluated in a range of voltages between operating voltage and low voltages down to 23 V. Polarity correction factor was measured for PPC05 chambers but not for the Razor Nano chamber.

## 3 RESULTS

### 3.1 Calorimeter measurements

Impurity correction factors obtained were 1.0001 for 100 MeV and 1.0002 for 160 MeV and 226 MeV. Heat loss correction factors were 1.0023 for 13 × 13 spots to 21 × 21 spots maps and 1.0015 for the 41 × 41 spots map. Absorbed dose to graphite results at a depth of 4.3 cm in water are summarized in Table 2. Specific heat capacity values ranged from 723.3 J·kg^−1^·K^−1^ to 724.8 J·kg^−1^·K^−1^ depending on calorimeter operating temperature during each irradiation.

**TABLE 2:** Temperature increments and resultant absorbed dose to graphite values measured with the portable calorimeter. For each irradiation condition the average of several irradiations is shown. For temperature increments, standard deviation at *k =* 1 is shown. For absorbed dose to graphite, combined standard uncertainty at *k =* 1 is shown.

Energy (MeV)	*map* (X x Y spots)	Clinical center	Residual range (g·cm^2^)	ΔT (K)	Dg (Gy)
100	13 × 13	1	3.44	$\left(1.0481\pm 0.0002\right)\times 10^{-3}$	0.762 ± 0.003
	14 × 14	2	3.41	$\left(1.0851\pm 0.0003\right)\times 10^{-3}$	0.787 ± 0.003
160	18 × 18	1	13.31	$\left(1.6982\pm 0.0005\right)\times 10^{-3}$	1.234 ± 0.004
		2	13.28	$\left(1.6209\pm 0.0004\right)\times 10^{-3}$	1.175 ± 0.004
	41 × 41	2	13.28	$\left(2.352\pm 0.002\right)\times 10^{-3}$	1.704 ± 0.006
226	21 × 21	1	27.74	$\left(2.3229\pm 0.0009\right)\times 10^{-3}$	1.686 ± 0.006
		2	27.67	$\left(2.382\pm 0.002\right)\times 10^{-3}$	1.680 ± 0.006

An example of temperature variation measured with the calorimeter core Wheatstone bridge can be seen in Figure 1 for a series of six runs, each run represents an irradiation of a 5 cm x 5 cm maps of 160 MeV.

**FIGURE 1:**
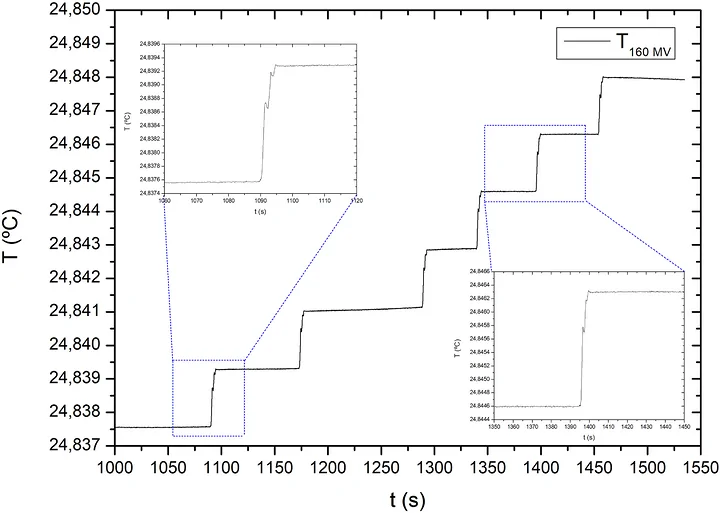
Example of a series of 160 MeV 5 cm x 5 cm beam irradiations. Top left and bottom right subplots show zoomed individual irradiations, where the three bursts of each irradiation can be identified.

### 3.2 Monte Carlo conversion factors

A summary of beam parameters for our Monte Carlo model can be seen in Table 4. These parameters are the same for each of the beamlets in a map. Comparison of measured and simulated IDD curves is shown in Figure 2. Relative local differences calculated as the difference between calorimeter and PPC05 derived absorbed dose to water values relative to PPC05 derived absorbed dose to water for full maps and at measuring depths different from reference depth are shown in Table 3.

**FIGURE 2:**
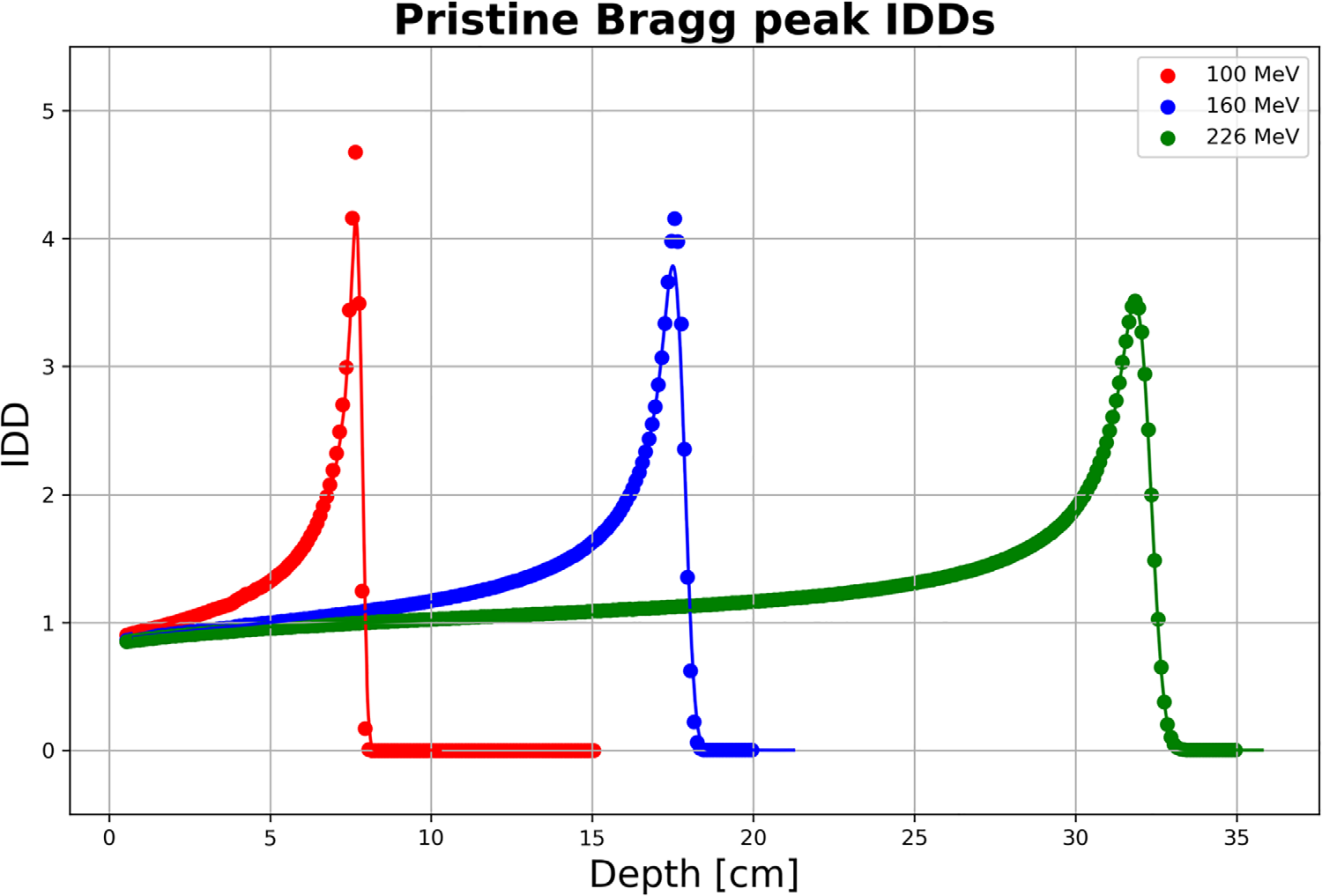
Experimental (line) and simulated (dot) Pristine Bragg peak IDDs curves for the IBA Stingray chamber as function of depth in water. Each energy curve has been normalized to its corresponding reference depth, as selected in center 2 (2 cm, 5 cm, and 8 cm, for 100, 160 and 226 MeV, respectively).

**TABLE 3:** Absorbed dose to water values measured at depths in water different from the calorimeter effective point of measurement. Comparison of values obtained from PPC05 ion chamber, $D_{w}{\left(z_{ref}\right)}_{PPC05}$, and determined with our calorimeter, $D_{w}{\left(z_{ref}\right)}_{cal}.$

E (MeV)	*map* (X x Y spots)	$z_{w}$(cm)	$D_{w}{\left(z_{ref}\right)}_{PPC05}$	$D_{w}{\left(z_{ref}\right)}_{cal}$	$\frac{D_{w}\left(cal\right)-D_{w}\left(PPC05\right)}{D_{w}\left(PPC05\right)}\times 100$
100	14 × 14	2	0.715 ± 0.012	0.699 ± 0.003	−2.2%
160	18 × 18	5	1.304 ± 0.022	1.311 ± 0.006	+0.5%
	41 × 41	5	1.903 ± 0.032	1.910 ± 0.009	+0.3%
226	21 × 21	8	1.856 ± 0.032	1.876 ± 0.009	+1.1%

**TABLE 4:** Beam parameters for the benchmarked model. Spatial pencil beam FWHM is assumed symmetrical in X and Y axis and is specified at source plane which in our case is placed at 45.5 cm from phantom surface.

E (MeV)	Rectangular momentum spread (MeV/c)	Spatial FWHM (cm)	Beam divergence (mrad)
100	0.002	1.025	5.5
160	0.0016	0.731	4.0
226	0.001	0.65	2.2

Graphite to water Monte Carlo conversion factors for the single energy layer maps considered in this work are shown in Table 5.

**TABLE 5:** MC factors for each dose map simulated, obtained by calculating the quotient between the water phantom body dose map and the calorimeter dose map at a depth in water of 4.3 cm. Uncertainties shown are evaluated as type A.

E (MeV)	*map* (X x Y spots)	$C_{g,w}\left(\;z_{w}=4.3\;cm,map,E\right)$
100	13 × 13	1.087 ± 0.003
	14 × 14	1.092 ± 0.003
160	18 × 18	1.096 ± 0.003
	41 × 41	1.100 ± 0.003
226	21 × 21	1.090 ± 0.003

Absorbed dose to water obtained by multiplying results in Table 2 by the factors tabulated in Table 5 are shown in Table 6.

**TABLE 6:** Absorbed dose to water values measured with our calorimeter at a depth of 4.3 cm in water.

E (MeV)	*map* (X x Y spots)	Center	Residual range (g·cm^2^)	Dw (Gy)
100	13 × 13	1	3.44	0.828 ± 0.004
	14 × 14	2	3.41	0.859 ± 0.004
160	18 × 18	1	13.31	1.353 ± 0.006
		2	13.28	1.288 ± 0.006
	41 × 41	2	13.28	1.874 ± 0.009
226	21 × 21	1	27.74	1.837 ± 0.009
		2	27.67	1.831 ± 0.009

### 3.3 Ion chamber measurements

A summary of ionization chamber results for obtaining $k_{Q}$ is shown in Table 7. No anomalous behavior^24^ was detected in the measurement of the Jaffé plot and therefore the saturation correction factor was calculated from the linear expression. An example of Jaffé plot is shown in Figure 3.

**TABLE 7:** Cobalt 60 calibration coefficients, polarity and saturation correction factors obtained for the chambers considered in this study.

Ion chamber	$N_{D,w,Q_{0}}$ (Gy/C)	E (MeV)	$k_{pol}$	$k_{s}$
PPC05#475	5.828×10^8^	226	1.001	1.002
PPC05#1277	5.934×10^8^	160	1.000	1.001
PPC05#1496	5.688×10^8^	226	1.002	1.003
RAZOR NANO#17475	1.004×10^10^	226	–	1.001

**FIGURE 3:**
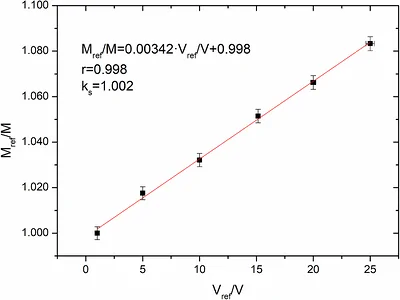
Normalized chamber response versus normalized polarization voltage for chamber PPC05‐475 in a 226 MeV proton beam (Jaffé plot). The intercept of the linear fit is the inverse of the saturation factor for an operating voltage of 500 Volt.

### 3.4 Beam quality correction factors

Beam quality correction factors determined in this work are shown in Tables 8 and 9. For PPC05 chamber, results are shown graphically together with results compiled from other works in Figure 4.

**TABLE 8:** Beam quality correction factors for PPC05 chamber as a function of residual range in water. For each range, the average of the three serial numbers is shown.

	Residual range (g·cm^−2^)	$k_{Q}$		
E (MeV)		SN	individual	average
100	3.4	475	0.998	0.997 ± 0.004
		1496	1.004	
		1277	0.989	
160	13.3	475	0.999	0.999 ± 0.003
		1496	1.004	
		1277	0.993	
226	27.7	475	0.999	0.999 ± 0.003
		1496	1.005	
		1277	0.993	

**TABLE 9:** Beam quality correction factors for Razor Nano chamber as a function of range in water.

E (MeV)	Residual range (g·cm^−2^)	$k_{Q}$
160	13.3	1.009
226	27.7	1.011

**FIGURE 4:**
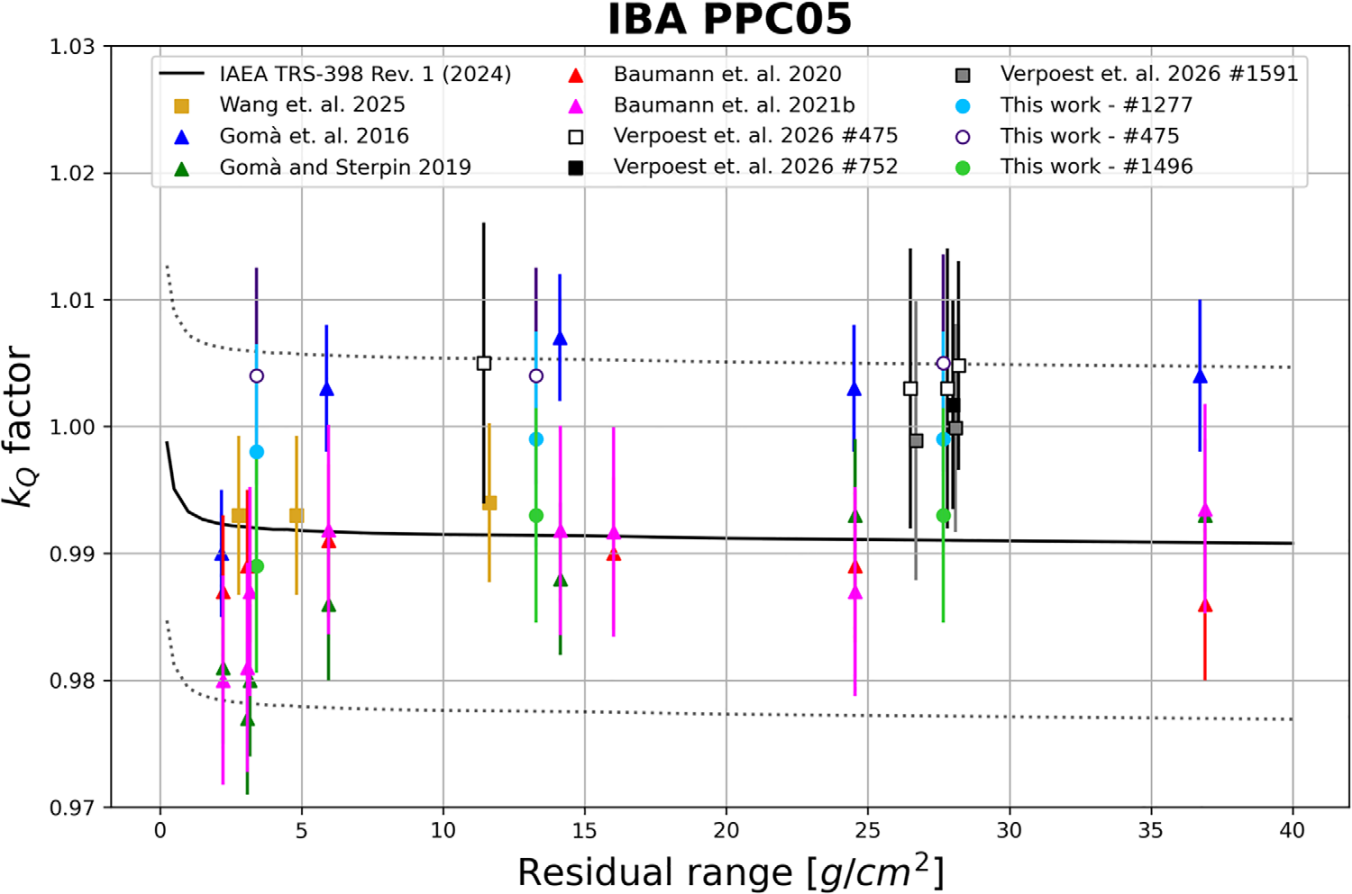
Beam quality correction factors $k_{Q}$ for the IBA PPC05 chamber as a function of residual range. For comparison purposes, the data found in TRS‐398 Rev. 1 for this chamber has also been plotted as well as its uncertainty range (k=1) as dotted lines. Note that triangles represent MC simulated factors and squares represent those calculated by water‐based calorimetry. Our data can be found represented as circles. The PPC05 chamber with serial number #475 was also used by Verpoest et al 2026, so we found fitting to plot our data accordingly.

### 3.5 Uncertainty budget

Uncertainty sources and overall standard uncertainty for beam quality correction factor determination can be summarized in Table 10.

**TABLE 10:** Different components of beam quality correction factor standard uncertainty (*k =* 1).

Component	Type A	Type B
**Specific heat capacity**		0.20
**Impurities correction**		0.06
**Monte Carlo conversion factor**		0.63
**Heat loss correction**		0.15
**Temperature readout**	0.09	0.17
**Beam reproducibility**	0.10	
**Absorbed dose to water Combined standard uncertainty**	0.71	
**Chamber positioning**		0.15
**Chamber reading**	0.05	0.10
**Beam reproducibility**	0.10	
**Saturation effect**		0.20
**Charge Combined standard Uncertainty**	0.29	
**Calibration coefficient in cobalt 60 beam combined uncertainty**	0.40	
**Beam Quality Correction factor Combined standard uncertainty**	0.87	

## 4 DISCUSSION

The portable graphite calorimeter showed excellent temperature stability during both measurement campaigns. Temperature increase per delivered map is reproducible within output stability. Instantaneous temperature reading can follow the dose delivered by each spot without significant heat losses, even for the delivery pattern of the blind golfer approach. This holds even for the 10 cm x 10 cm field which takes longer to be delivered.

The system setup can be completed onsite in less than 90 min and no more than 3 to 7 repetitions per beam quality were required to produce a precise measurement. Temperature increments measured by the calorimeter exhibited a relative standard deviation below 0.1% after just 5 repetitions. This means that room occupancy can be kept low and not interfere in clinical routine.

Monte Carlo dose to graphite to dose to water conversion factor variation with beam quality shows a low energy dependence. These values are similar to those obtained for another graphite calorimeter in the literature^16^, ^25^ although in the latter case the authors separated the conversion factor into several factors ($k_{gap},\;s_{w,g},\;k_{fl},\;k_{z,cal}$). The agreement of the relative depth dose for pristine beams and for single layer maps between PPC05 and calorimeter was below 2.2% for all energies, with the lowest energy pristine beam presenting the largest discrepancy.. These differences are compatible with uncertainties.

We can see the comparison between the average $k_{Q}$ factors obtained in this work for the PPC05 chambers and those found in the literature (obtained by Monte Carlo simulation^2^, ^3^, ^4^, ^5^ or by water calorimetry^12^, ^13^) yield similar results, within 1%. Our average results for beam quality correction factors are greater than those recommended by TRS‐398 but consistent with those found in the literature for similar measurement depths. This is consistent with prior findings by other authors which have raised the impact of chamber reference point positioning convention in Monte Carlo calculated beam quality correction factors.^26^

In the case of the IBA Razor Nano chamber, the measured $k_{Q}$ factors were 1.009 (13.3 g·cm^−2^) and 1.011 (27.7 g·cm^−2^). These values are small compared to those tabulated in TRS‐398 for other cylindrical chambers but similar to the smaller active volume cylindrical chamber tabulated. This suggests that fluence perturbation plays an important role added to the material caused energy dependence in the amount of correction required by cylindrical ion chamber‐based proton dosimetry. The chamber exhibited a polarity effect close to 1% and therefore it was decided to use positive polarity only. Noise to signal ratio was kept below 0.04%. Also, stability and reproducibility of positive polarity values were investigated and both same day and day to day differences were below 0.4%. Consequently, $k_{Q}$ factor uncertainty for this chamber should be increased accordingly. Although this data is useful, it is recommended that more studies be devoted to the determination of $k_{Q}$ factors, both by considering more chamber units and more energies and further investigating chamber polarity effect.

The uncertainty budget detailed in Table 10 demonstrates that the $k_{Q}$ determination with our device has a final combined standard uncertainty of about 1% (k=1). The dominant components arise from 60‐Co calibration (0.4%) and from Monte Carlo conversion factor (0.6%). Regarding the calorimeter, the dominant component of uncertainty is associated with the specific heat capacity of graphite (0.20%). This global balance places portable graphite calorimetry at a similar level as the water calorimetry, with the advantage of its portability and relatively easy and fast set up allowing for low occupation time in clinical facilities.

## 5 CONCLUSIONS

In this work, the performance and operational feasibility of a portable graphite calorimetry system under pulsed pencil beam scanning proton beams have been demonstrated. Throughout the measurement campaigns, no significant dosimetric biases were observed against ionization chamber reference dosimetry. No significant heat losses were observed, validating the robustness of the thermal insulation of the calorimeter and core temperature control in real clinical environments. The portable graphite calorimeter allows for absorbed dose to water determination with an uncertainty of 0.7% and, by directly comparing its response with the response of an ionization chamber, for beam quality correction factor determination with an uncertainty of 1%.

Beam quality correction factors agreed with other experimental values found in literature for the PPC05 chamber and confirmed the importance of a clear definition of chamber positioning in reference dosimetry. This also stresses the importance of producing experimental data to validate simulated data. Data presented for the razor nano chamber, although limited, contribute to future dosimetric studies.

The system has an easy and fast set up and can produce repeatable results after a small number of samples. Its thermal stability allows for a low sampling time. These reasons support the validity of this portable calorimeter as an efficient tool both for conducting independent absolute dose audits in proton therapy centers and for the in situ determination of institution‐specific beam quality correction factors, contributing to the generation of dosimetric reference data.

## AUTHOR CONTRIBUTIONS

Nicolás Gómez‐Fernández designed, assembled and characterized the calorimeter, carried out the experimental campaigns at protontherapy centres and made the data analysis. Eloy González García performed the Monte Carlo simulations. Séverine Rossomme, Lies Verpoest, Juan Antonio Vera participated in the experimental campaigns. Faustino Gómez and Alejandro Mazal reviewed the manuscript and Diego M González‐Castaño coordinated the Calorimeter development project, helped with the Monte Carlo Simulations for conversion factors and helped with the experimental campaign and data analysis. All authors contributed to the discussion of results.

## FUNDING INFORMATION

This work was supported by Instituto de Salud Carlos III [Grant number: PI19/00263].

## CONFLICT OF INTEREST STATEMENT

The authors declare no conflicts of interest

## ETHICAL APPROVAL

Ethics approval was not required for this study, as no patient data were involved.

## Data Availability

The data that support the findings of this study are available from the authors upon reasonable request
